# Development and validation of an interpretable machine learning model integrating baseline multimodal CT perfusion and clinical data for predicting 9-month functional outcomes in acute ischemic stroke

**DOI:** 10.3389/fneur.2026.1877613

**Published:** 2026-08-26

**Authors:** Guizhi Zhang, Yuanli Zhang, Xin Chen, Bo Han, Xiang Li, Yang Wang, Bo Jiang

**Affiliations:** 1 Department of Medical Technology, Chongqing Three Gorges Medical College, Chongqing, China; 2 Research Centre of Oral Materials and Technology, Chongqing Three Gorges Medical College, Chongqing, China; 3 Department of Radiology, People's Hospital Affiliated to Chongqing Three Gorges Medical College, Chongqing, China; 4 Radiology of Neurology, Chongqing University Three Gorges Hospital, Chongqing, China

**Keywords:** acute ischemic stroke, CT perfusion, functional outcome, machine learning, predictive model, SHAP, XGBoost

## Abstract

**Background and purpose:**

Accurate early prediction of long-term functional outcomes in acute ischemic stroke (AIS) remains challenging. We aimed to develop and validate an interpretable machine learning model integrating baseline multimodal CT perfusion and clinical data to predict 9-month poor functional outcome [modified Rankin Scale (mRS) 3–6] in AIS patients undergoing endovascular or surgical intervention.

**Methods:**

This retrospective study included 371 AIS patients who underwent endovascular or surgical intervention. We integrated clinical variables, laboratory markers, CT perfusion parameters (Tmax, cerebral blood flow, and cerebral blood volume), and quantitative CT density measurements [Hounsfield units on the affected and healthy sides, normalized water uptake (NWU), Alberta Stroke Program Early CT Score (ASPECT score)]. Feature selection was performed using LASSO regression followed by SHAP-based refinement. Four algorithms—Logistic Regression, Random Forest, Support Vector Machine, and XGBoost—were compared. Model performance was evaluated using area under the receiver operating characteristic curve (AUC), sensitivity, specificity, F1-score, Brier score, calibration curves, and decision curve analysis (DCA). SHAP analysis was used to enhance model interpretability.

**Results:**

Eleven predictors were selected by LASSO; after SHAP-based refinement, ten remained in the final model. The XGBoost model achieved the best performance, with an AUC of 0.956 (95% CI 0.917–0.995), sensitivity of 0.932, specificity of 0.897, F1-score of 0.938, and Brier score of 0.0713 (95% CI 0.0438–0.1135). SHAP analysis identified door-to-intervention interval (DTI) (mean SHAP = 0.95) as the most influential predictor, followed by HU_affected (0.88), CBV < 42% (0.82), NLR (0.78), blood glucose (0.74), age (0.70), gender (0.68), ASPECT score (0.66), HU_healthy (0.50), and NWU (0.48). Calibration curves demonstrated excellent agreement between predicted and observed outcomes, and DCA confirmed superior net benefit of the XGBoost model across clinically relevant thresholds (0.01–0.60).

**Conclusion:**

We developed an interpretable XGBoost-based model integrating baseline multimodal CT perfusion and clinical data that accurately predicts 9-month functional outcomes in AIS patients undergoing endovascular or surgical intervention. DTI, HU_affected, and CBV < 42% emerged as the dominant predictors, highlighting the prognostic importance of time-to-reperfusion and baseline tissue injury. After external validation, this model may serve as a practical tool for early risk stratification and individualized rehabilitation planning.

## 1 Introduction

Stroke remains a leading cause of death and long-term disability worldwide (1). Acute ischemic stroke (AIS) accounts for the majority of all stroke cases and imposes a substantial burden on patients, families, and healthcare systems globally (2). Even with timely reperfusion therapies—including intravenous thrombolysis and endovascular thrombectomy—a considerable proportion of patients fail to achieve favorable functional recovery (3). The modified Rankin Scale (mRS) is the most widely used outcome measure in stroke clinical trials and routine practice, with scores of 3 to 6 generally defined as poor functional outcome (4). Accurate early prediction of 9-month functional outcomes is therefore essential for risk stratification, clinical decision-making, and personalized rehabilitation planning (5).

Traditional approaches to stroke outcome prediction have relied heavily on clinical scales such as the National Institutes of Health Stroke Scale (NIHSS), along with logistic regression models incorporating a limited set of clinical and demographic variables. While these tools are practical at the bedside, they have inherent limitations. NIHSS is a semi-quantitative clinical assessment subject to inter-observer variability (6), and conventional regression models assume linear relationships between predictors and outcomes—an assumption that may not hold given the complex, non-linear interactions among stroke-related pathophysiological processes (7). Moreover, traditional models often fail to fully leverage the rich information available from multimodal imaging and laboratory data (8).

In recent years, machine learning (ML) has emerged as a powerful alternative for clinical outcome prediction. Unlike conventional statistical methods, ML algorithms can capture complex, non-linear relationships and interactions among high-dimensional clinical, laboratory, and imaging features without requiring pre-specified model structures. Among various ML algorithms, extreme gradient boosting (XGBoost) has gained particular attention in stroke research due to its superior predictive performance, built-in regularization to prevent overfitting, and robust handling of heterogeneous data. A systematic review and meta-analysis of AI models for stroke prognosis prediction reported that ML models achieved good predictive accuracy for ischemic stroke outcomes (9). Multiple recent studies have further validated the superiority of XGBoost over traditional logistic regression in predicting stroke outcomes. Chung et al. developed an XGBoost-based model using age, fasting glucose, and NIHSS scores to predict three-month functional outcomes after AIS, achieving an AUC of 0.8595 (10). Lee et al. reported that ML algorithms demonstrated proficient prediction for the 3-month functional outcome in AIS patients, with the SHAP method providing interpretable insights into how critical features contribute to model predictions (11). Another study focusing on AIS-LVO patients undergoing mechanical thrombectomy within an extended therapeutic window found that XGBoost emerged as the most effective model for predicting prognosis, with a validation AUC of 0.93 (12). Ozkara et al. utilized imaging parameters alone in ML models to predict functional outcomes in AIS patients with anterior circulation large vessel occlusions (13). These findings collectively support the potential of XGBoost as a robust tool for stroke prognostication.

Beyond algorithm selection, the choice of predictors is equally critical for model performance. Recent evidence has highlighted the prognostic value of multimodal CT perfusion imaging parameters in AIS. Parameters such as Tmax (time-to-maximum of the residue function), cerebral blood flow (CBF), and cerebral blood volume (CBV) provide quantitative assessments of ischemic penumbra and infarct core—information that is not captured by clinical scales alone. Studies have shown that CT perfusion-derived parameters can reliably predict functional outcomes and hemorrhagic transformation. In particular, a CBV threshold of < 42% of normal has been identified as a marker of irreversibly injured ischemic core. Lakhani et al. demonstrated that rCBV < 42% is negatively associated with poor functional outcomes at discharge (mRS ≥ 3) in anterior circulation large vessel occlusion stroke (aOR = 0.97, *p* < 0.05) (14). Additionally, automated ASPECTS-based net water uptake (NWU) has recently been shown to provide complementary prognostic information to conventional CT perfusion parameters, offering an alternative imaging biomarker for patient selection (12).

Systemic inflammatory markers have also emerged as important prognostic factors in AIS. The neutrophil-to-lymphocyte ratio (NLR), a readily available and cost-effective biomarker of systemic inflammation, has been consistently associated with stroke severity, unfavorable functional outcomes, and mortality (9). Recent studies have further confirmed that NLR independently contributes to unfavorable prognosis in both general and elderly AIS populations, and that higher NLR levels at admission may lead to unfavorable 90-day outcomes in patients undergoing endovascular therapy.

While numerous ML models have been developed for short-term (e.g., 3-month) outcome prediction, fewer studies have focused on longer-term functional outcomes such as 9-month mRS. Furthermore, many existing models rely on either clinical variables alone or imaging parameters alone, without fully integrating both domains. The interpretability of ML models—a key barrier to clinical adoption—has also been insufficiently addressed in prior work. The SHapley Additive exPlanations (SHAP) method offers a unified framework for quantifying each feature’s contribution to model predictions, thereby enhancing model transparency and clinical trustworthiness (11). Studies employing SHAP analysis in stroke outcome prediction have identified imaging and clinical parameters as key determinants of outcome (12).

In this study, we aimed to develop and validate an interpretable machine learning model integrating multimodal baseline CT perfusion and clinical data to predict 9-month poor functional outcome in patients with acute ischemic stroke undergoing endovascular or surgical intervention. We further sought to identify the most informative predictors among clinical, laboratory, and imaging parameters, and to compare the predictive performance of multiple algorithms, with the hypothesis that an XGBoost model incorporating multimodal data would achieve superior discriminative ability and calibration.

## 2 Methods

### 2.1 Study design and participants

This retrospective study included patients with acute ischemic stroke who underwent multimodal CT perfusion imaging and received endovascular or surgical intervention at Chongqing University Three Gorges Hospital between January 2024 and June 2025. For this analysis, we included only patients who had completed their 9-month functional outcome assessment by the time of data lock (March 2026). We enrolled patients who met the following criteria: (1) age 18 years or older; (2) confirmed diagnosis of acute ischemic stroke; (3) complete admission laboratory data and pretreatment CT perfusion imaging available; and (4) 9-month functional outcome documented using the modified Rankin Scale (mRS). We excluded patients with pre-stroke mRS ≥ 3 (indicating prior functional dependence), intracranial hemorrhage or other non-ischemic etiologies, and those with incomplete imaging or clinical records. The final analysis included 371 patients. The Institutional Review Board of Chongqing University Three Gorges Hospital approved this study and waived the requirement for informed consent given its retrospective design.

### 2.2 Data collection

We extracted demographic and clinical data from electronic medical records, including age, sex, admission blood glucose, and complete blood count parameters. The neutrophil-to-lymphocyte ratio (NLR) was derived by dividing the absolute neutrophil count by the absolute lymphocyte count. All laboratory measurements were obtained within 24 h of admission.

Trained neurologists who were blinded to imaging findings assessed the 9-month functional outcome using the modified Rankin Scale (mRS) through standardized telephone interviews or outpatient visits. We defined poor functional outcome as mRS 3–6 and good outcome as mRS 0–2, following conventional practice in stroke outcome research.

### 2.3 Imaging acquisition and processing

All patients underwent non-contrast CT and CT perfusion on admission using a 64-slice multi-detector CT scanner (SOMATOM Definition Edge, Siemens Healthineers, Erlangen, and Germany). We processed the perfusion data with RAPID (Rapid Processing of Perfusion and Diffusion, iSchemaView, Menlo Park, CA, United States), an automated software that applies a delay and dispersion corrected deconvolution algorithm to generate parametric maps. The software automatically produced Tmax, CBF, and CBV parametric maps. Volumes with Tmax > 6 s, Tmax > 10 s, and CBF < 30% were automatically generated. The volume with CBV < 42% was subsequently calculated from the CBV maps to define the ischemic core, following previously validated thresholds.

For density measurements, experienced neuroradiologists manually delineated regions of interest on non-contrast CT images. We recorded Hounsfield unit (HU) values from the affected hemisphere (HU_affected) and the corresponding mirror region in the healthy hemisphere (HU_healthy), and calculated the Alberta Stroke Program Early CT Score (ASPECT score) for each patient. The normalized water uptake (NWU) was computed as:

NWU=(1−HU_affected/HU_healthy)×100%

We also recorded the door-to-intervention interval (DTI), defined as the time from arrival at the emergency department of our comprehensive stroke center to the initiation of the endovascular or surgical procedure. For patients transferred from referring hospitals, we used the arrival time at our center—not the time at the referring hospital—as the start point, consistent with the standard definition of “door-to-puncture” time in stroke guidelines.

### 2.4 Feature selection

We started with LASSO regression using 10-fold cross-validation to screen variables from all candidates. LASSO applies an L1 penalty that shrinks coefficients of irrelevant predictors to zero, effectively selecting a compact set of candidates. We chose the *λ*.min value—the penalty level that minimized cross-validated deviance—and retained all variables with non-zero coefficients at that λ for subsequent steps.

To further trim redundant or collinear features, we built an initial XGBoost model using the LASSO-selected variables and calculated SHAP (SHapley Additive exPlanations) values for each feature. SHAP provides a unified framework for quantifying each predictor‘s contribution to the model’s predictions; we used the mean absolute SHAP value as a global importance metric and sequentially excluded variables with marginal contributions—specifically, those scoring below a prespecified threshold (mean |SHAP| < 0.30). In cases where two variables showed high collinearity (Pearson correlation coefficient > 0.7), the variable with lower SHAP importance was excluded. The remaining variables were retained for subsequent model development and comparison across algorithms.

### 2.5 Model development

We compared four classification algorithms using the final set of predictors: Logistic Regression (LR): A linear model with L2 regularization, included as a traditional baseline. Random Forest (RF): An ensemble of decision trees that averages predictions to reduce overfitting and capture non-linear patterns. Support Vector Machine (SVM): A classifier that finds the hyperplane maximizing class separation. We used a linear kernel for interpretability; additional analyses with an RBF kernel were performed in sensitivity analyses. XGBoost (XGB): A gradient-boosted tree algorithm that builds trees sequentially, with each new tree correcting errors from the previous ones. It includes built-in regularization and handles missing data well.

We implemented all models in Python 3.9 using scikit-learn 1.1 and XGBoost 1.7. Hyperparameters were tuned via grid search with 5-fold cross-validation on the training set, optimizing for AUC.

### 2.6 Training and validation

We randomly split the cohort into training and validation sets at a 7:3 ratio. The training set was used for feature selection, model development, and hyper parameter tuning. The validation set was kept untouched until the final evaluation to provide an unbiased estimate of generalizability. All included patients had complete data for the candidate predictors; therefore, no imputation was needed for the primary analysis.

### 2.7 Performance evaluation

We assessed model performance in the validation set using several metrics: Discrimination: Measured by the AUC, with 95% confidence intervals estimated via 1,000 bootstrap resamples. Classification accuracy: Reported sensitivity, specificity, positive predictive value, negative predictive value, and F1-score. We determined the classification threshold using the Youden index from the training set. Calibration: Evaluated with the Brier score (mean squared difference between predicted probabilities and observed outcomes). Lower values indicate better calibration; scores below 0.10 are generally considered excellent. We also plotted calibration curves with 5 quantile-based bins for visual inspection. Bootstrap resampling provided 95% CIs for Brier scores. Agreement between predicted and observed outcomes was further assessed using Cohen’s Kappa. Clinical utility: Assessed via decision curve analysis (DCA), which estimates the net benefit of using the model across a range of threshold probabilities, compared with “treat-all” and “treat-none” strategies.

### 2.8 Model interpretation

For the best-performing model (XGBoost), we used SHAP to explain its predictions. We applied the TreeExplainer algorithm to calculate SHAP values for each feature in the validation set. The mean absolute SHAP value served as a global importance metric, and we generated beeswarm summary plots to visualize the direction and magnitude of each predictor’s effect—red points indicate higher feature values, blue points lower values.

### 2.9 Statistical analysis

We summarized continuous variables as median with interquartile range (IQR) and categorical variables as frequencies with percentages. To compare training and validation sets, we used the Wilcoxon rank-sum test for continuous variables and the chi-square or Fisher‘s exact test for categorical variables, as appropriate. LASSO regression was run in R 4.2 using the glmnet package with binomial family and 10-fold cross-validation. Machine learning models were built in Python 3.9, using scikit-learn 1.1 for LR, RF, and SVM, and the XGBoost library 1.7 for the gradient-boosted model. Calibration and DCA were implemented with custom Python scripts drawing on sklearn.calibration and sklearn.metrics functions. All tests were two-sided; we considered *p* < 0.05 statistically significant.

## 3 Results

### 3.1 Baseline characteristics

**Figure 1:**
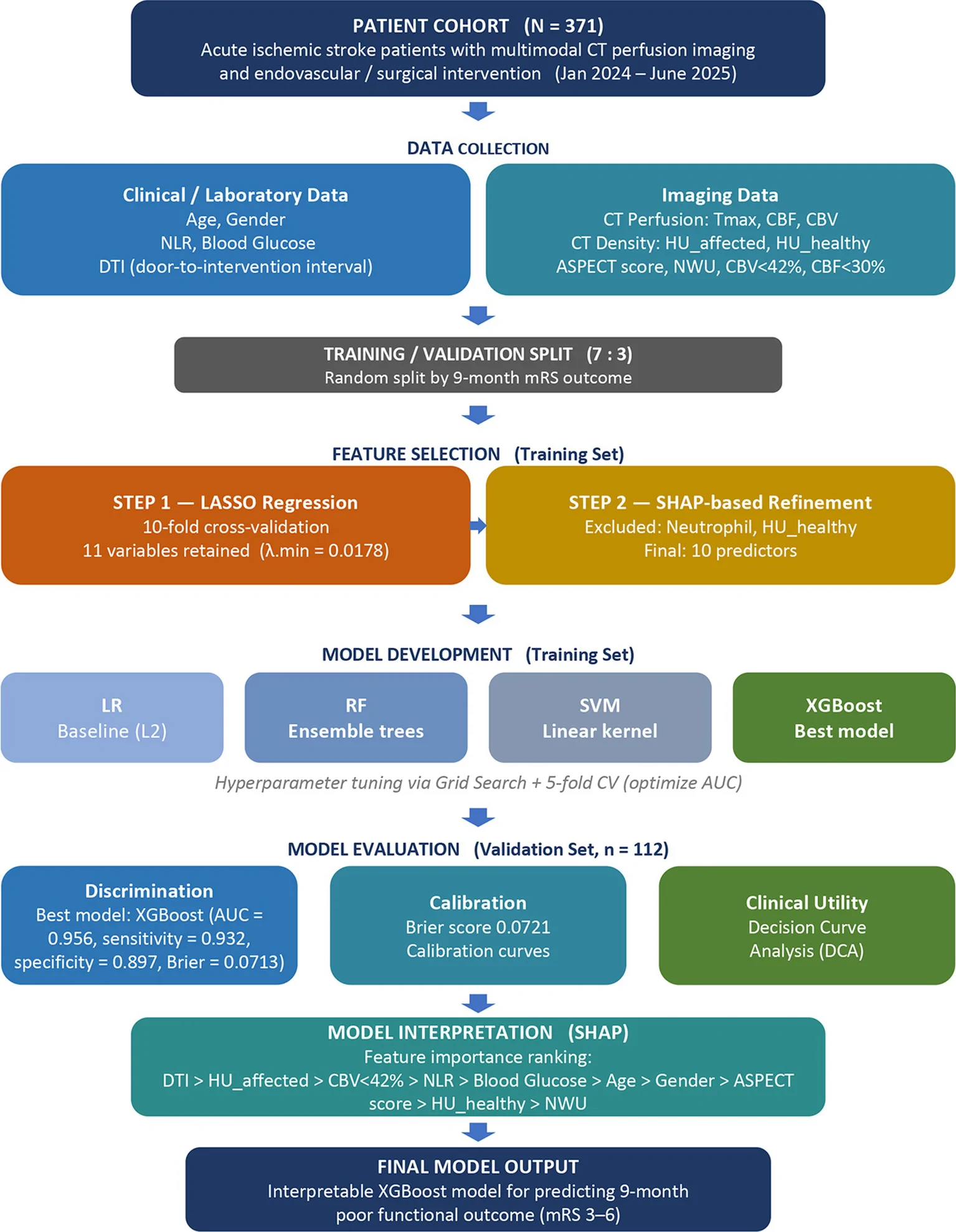
Study flowchart. AIS, acute ischemic stroke; NLR, neutrophil-to-lymphocyte ratio; DTI, door-to-intervention interval; HU, Hounsfield unit; NWU, normalized water uptake; ASPECT, Alberta Stroke Program Early CT Score; CBF, cerebral blood flow; CBV, cerebral blood volume; LASSO, least absolute shrinkage and selection operator; SHAP, SHapley Additive exPlanations; LR, logistic regression; RF, random forest; SVM, support vector machine; XGB, XGBoost; CV, cross-validation; AUC, area under the receiver operating characteristic curve; DCA, decision curve analysis.

The patient selection and study workflow are illustrated in Figure 1. A total of 371 patients with acute ischemic stroke who met the inclusion criteria were enrolled in this retrospective cohort study. Of these, 259 (69.8%) were assigned to the training set and 112 (30.2%) to the validation set through random split at a 7:3 ratio. Among patients who met the imaging and clinical inclusion criteria during the study period, 12 patients (3.1%) were lost to 9-month follow-up and were excluded, leaving a final cohort of 371 patients with complete imaging, clinical, and outcome data for analysis. The training set was used for feature selection (LASSO regression followed by SHAP-based refinement), model development, and hyper parameter tuning, while the validation set was held out for final model evaluation.

The baseline characteristics of the study population, random split by training and validation sets, are summarized in Table 1. The median age of the overall cohort was 71.0 years (IQR 62.0–77.7), and 220 (59.3%) patients were male. The proportion of patients with poor 9-month functional outcome (mRS 3–6) was 55.8% (207/371) in the overall cohort, 51.7% (134/259) in the training set, and 65.2% (73/112) in the validation set.

**Table 1:** Baseline characteristics of the study population stratified by training and validation sets.

Variables	Total (*N* = 371)	Validation (*N* = 112)	Train (*N* = 259)	*P*
Poor outcome, *n* (%)				0.023
0	164 (44%)	39 (35%)	125 (48%)	
1	207 (56%)	73 (65%)	134 (52%)	
Gender, *n* (%)				0.258
1	220 (59%)	61 (54%)	159 (61%)	
2	151 (41%)	51 (46%)	100 (39%)	
Age, median (Q1–Q3)	71 (62–77.7)	71 (61.8–78.8)	71 (62.2–76.7)	0.641
Neutrophil, median (Q1–Q3)	5.78 (4.34–8.27)	5.75 (4.52–8.68)	5.85 (4.13–8.06)	0.305
Lymphocyte, median (Q1–Q3)	1.18 (0.82–1.64)	1.22 (1.01–1.83)	1.15 (0.75–1.56)	0.005
NLR, median (Q1–Q3)	4.54 (2.87–8.61)	4.50 (2.92–8.82)	4.71 (2.87–8.61)	0.770
Blood Glucose, median (Q1–Q3)	6.90 (5.70–8.70)	6.80 (5.70–8.45)	6.90 (5.70–8.75)	0.609
DTI, median (Q1–Q3)	130 (100–180)	130 (90.8–181.3)	130 (105–180)	0.814
ASPECT score, median (Q1–Q3)	6 (3–8)	5 (2.75–8)	6 (4–8)	0.018
HU_affected, median (Q1–Q3)	140.9 (74.6–225.2)	154.9 (79.0–231.8)	130.8 (73.9–220.1)	0.203
HU_healthy, median (Q1–Q3)	154.1 (77.6–260.3)	172.9 (81.7–261.5)	146.3 (77.0–253.6)	0.179
AHUAS, median (Q1–Q3)	33.10 (30.76–35.67)	32.71 (30.46–35.52)	33.33 (30.83–35.67)	0.172
AHUHS, median (Q1–Q3)	36.05 (33.33–37.80)	35.99 (33.86–37.43)	36.10 (33.27–37.93)	0.580
NWU, median (Q1–Q3)	6.59 (4.80–8.94)	7.12 (5.23–10.46)	6.48 (4.68–8.49)	0.015
Tmax > 10 s, median (Q1–Q3)	51 (12–110)	61 (24–134)	46 (10.5–98)	0.034
Tmax > 6 s, median (Q1–Q3)	146 (80–214)	146 (91.8–202.3)	146 (78–217.5)	0.736
CBF < 30%, median (Q1–Q3)	15 (0–55.5)	16 (0–70.3)	14 (0–48.5)	0.075
CBV < 42%, median (Q1–Q3)	20 (0–71.5)	27.5 (5–84)	18 (0–67.5)	0.028

Data are presented as median (Q1–Q3) for continuous variables and as *n* (%) for categorical variables. *P* values were calculated using the Wilcoxon rank-sum test for continuous variables and the chi-square test for categorical variables. IQR, interquartile range; NLR, neutrophil-to-lymphocyte ratio; DTI, door-to-intervention interval; HU, Hounsfield unit; AHUAS, average Hounsfield unit on the affected side; AHUHS, average Hounsfield unit on the healthy side; NWU, normalized water uptake; Tmax, time-to-maximum of the residue function; CBF, cerebral blood flow; CBV, cerebral blood volume.

Although statistically significant differences were observed in several baseline characteristics between the training and validation sets, including lymphocyte count (*p* = 0.005), ASPECT score (*p* = 0.018), NWU (*p* = 0.015), Tmax > 10 s (*p* = 0.034), and CBV < 42% (*p* = 0.028), the absolute magnitudes of these differences were clinically modest and likely attributable to random sampling variation and multiple comparisons. Other variables, including age, gender, NLR, blood glucose, DTI, HU_affected, HU_healthy, AHUAS, AHUHS, Tmax > 6 s, and CBF < 30%, were well balanced between the two sets (all *p* > 0.05).

### 3.2 Feature selection via LASSO regression

**Figure 2:**
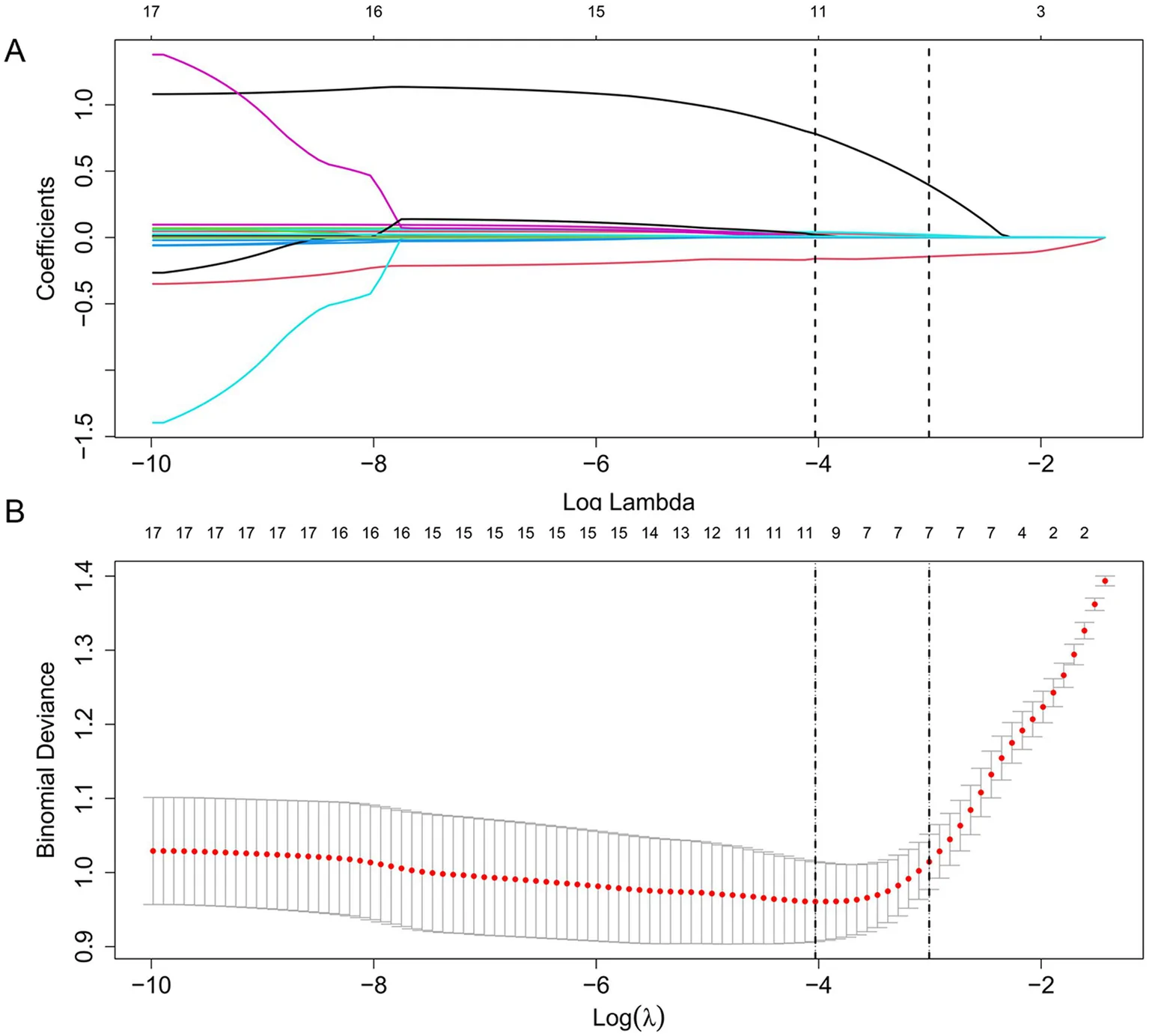
LASSO regression for feature selection. **(A)** Cross-validation curve. The left vertical dashed line indicates *λ*.min (0.0178), and the right dashed line indicates *λ*.1se. The *λ*.min value, which yielded the minimum cross-validated deviance, was selected for feature selection. **(B)** Coefficient profiles of the 11 candidate variables. Each curve represents the trajectory of a coefficient as the log(*λ*) penalty increases. Variables with non-zero coefficients at *λ*.min were retained for subsequent modeling.

LASSO regression with 10-fold cross-validation was applied to select the most relevant predictors from all candidate variables. The optimal penalty parameter *λ* was determined as the λ.min value (0.0178), which yielded the minimum cross-validated deviance (Figure 2A). At this λ value, 11 variables with non-zero coefficients were retained: Gender, Age, Neutrophil, NLR, Blood Glucose, DTI, ASPECT score, HU_affected, HU_healthy, NWU, and CBV < 42%. The coefficient profiles of these variables across the penalty path are shown in Figure 2B.

### 3.3 Further feature refinement and final model performance

To further refine the predictor set and prioritize clinically interpretable features, we calculated SHAP values based on an initial XGBoost model using the 11 LASSO-selected variables. A retention threshold of mean |SHAP| ≥ 0.30 was pre-specified to balance model parsimony with predictive performance, consistent with established practices in machine-learning-based prognostic modeling. All 11 LASSO-selected variables met this criterion, with mean SHAP values exceeding the pre-specified threshold.

However, examination of pairwise correlations among the candidate predictors revealed strong collinearity between Neutrophil and NLR (Pearson’s *r* = 0.79), which is expected given that NLR is derived directly from Neutrophil and Lymphocyte counts. To avoid multicollinearity and ensure model stability, Neutrophil was excluded from the final model despite meeting the SHAP threshold. The remaining 10 variables—DTI, HU_affected, CBV < 42%, NLR, Blood Glucose, Age, Gender, ASPECT score, HU_healthy, and NWU—were retained for final model development.

**Figure 3:**
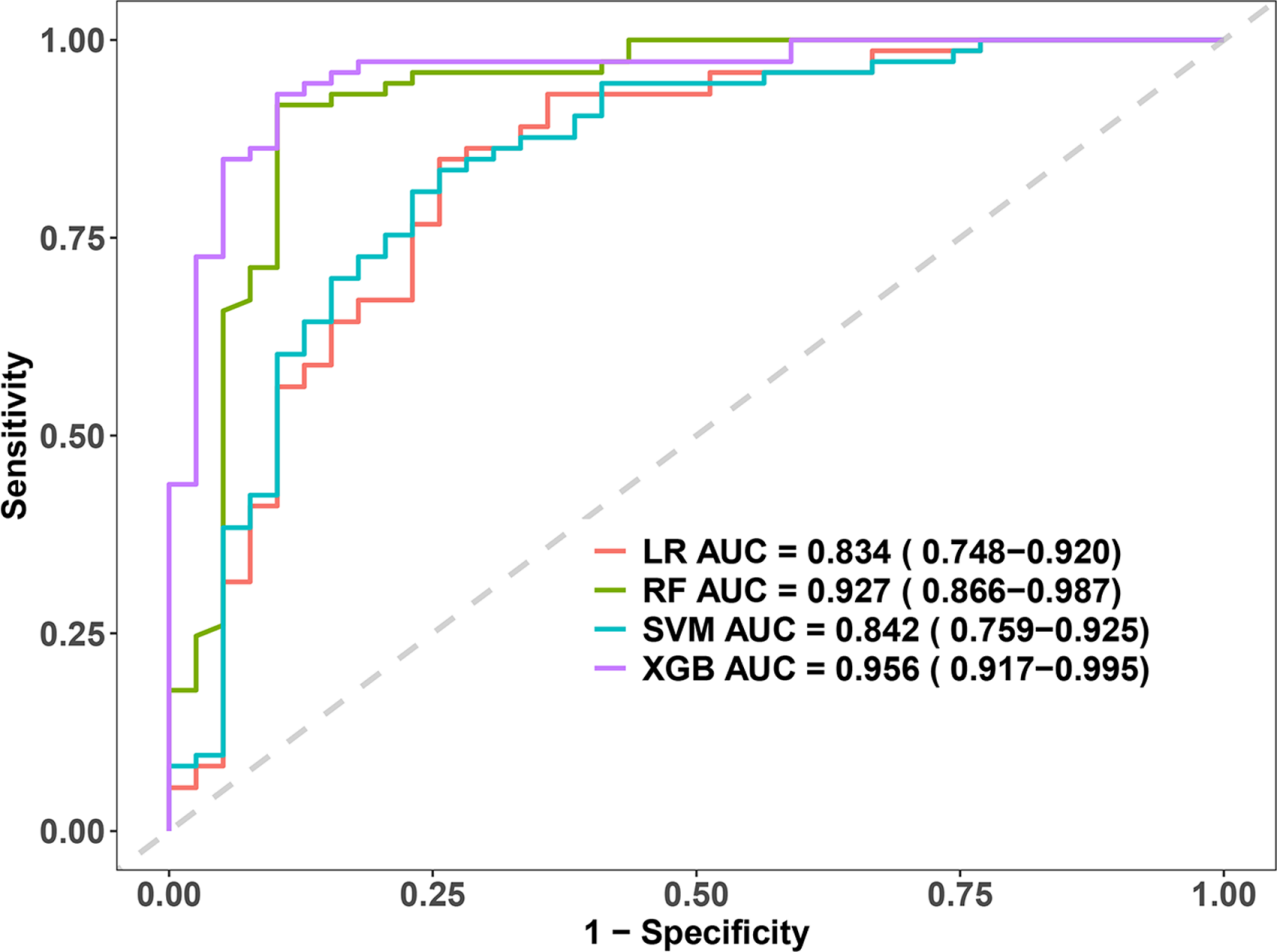
Receiver operating characteristic (ROC) curves of the four prediction models in the validation set. XGBoost demonstrated the highest AUC (0.956), followed by RF (0.927), SVM (0.842), and LR (0.834). The diagonal dashed line represents the reference line (AUC = 0.5).

Subsequently, all four classification algorithms (Logistic Regression, Random Forest, SVM, and XGBoost) were retrained and validated using this uniform set of ten predictors. The performance of the four models in the independent validation set is summarized in Table 2 and Figure 3.

**Table 2:** Performance of four prediction models in the validation set (*n* = 112).

Model	AUC (95% CI)	Sensitivity	Specificity	F1-score	Brier score (95% CI)
Logistic regression	0.834 (0.748–0.920)	0.932	0.641	0.877	0.1569 (0.1173–0.2025)
Random forest	0.927 (0.866–0.987)	0.918	0.897	0.931	0.0994 (0.0770–0.1385)
XGBoost	0.956 (0.917–0.995)	0.932	0.897	0.938	0.0713 (0.0438–0.1135)
SVM	0.842 (0.759–0.925)	0.828	0.744	0.774	0.5339 (0.4815–0.5903)

The ROC curves of the four models are presented in Figure 3. The XGBoost model achieved the highest AUC of 0.956 (95% CI 0.917–0.995), significantly outperforming Logistic Regression (0.834, 95% CI 0.748–0.920), Random Forest (0.927, 95% CI 0.866–0.987), and SVM (0.842, 95% CI 0.759–0.925).

In terms of sensitivity, Logistic Regression and XGBoost both ranked highest (0.932), followed by Random Forest (0.918), and SVM (0.828). For specificity, Random Forest and XGBoost both achieved the best performance (0.897), followed by SVM (0.744) and LR (0.641). The F1-score was highest for XGBoost (0.938), followed by RF (0.931), LR (0.877), and SVM (0.774). Regarding calibration, the XGBoost model yielded the lowest Brier score in the validation set (0.0713, 95% CI 0.0438–0.1135), indicating excellent agreement between predicted probabilities and observed outcomes. RF also showed acceptable calibration (0.0994, 95% CI 0.0770–0.1385), whereas LR (0.1569, 95% CI 0.1173–0.2025) and SVM (0.5339, 95% CI 0.4815–0.5903) exhibited poor calibration.

### 3.4 Feature importance and clinical utility

**Figure 4:**
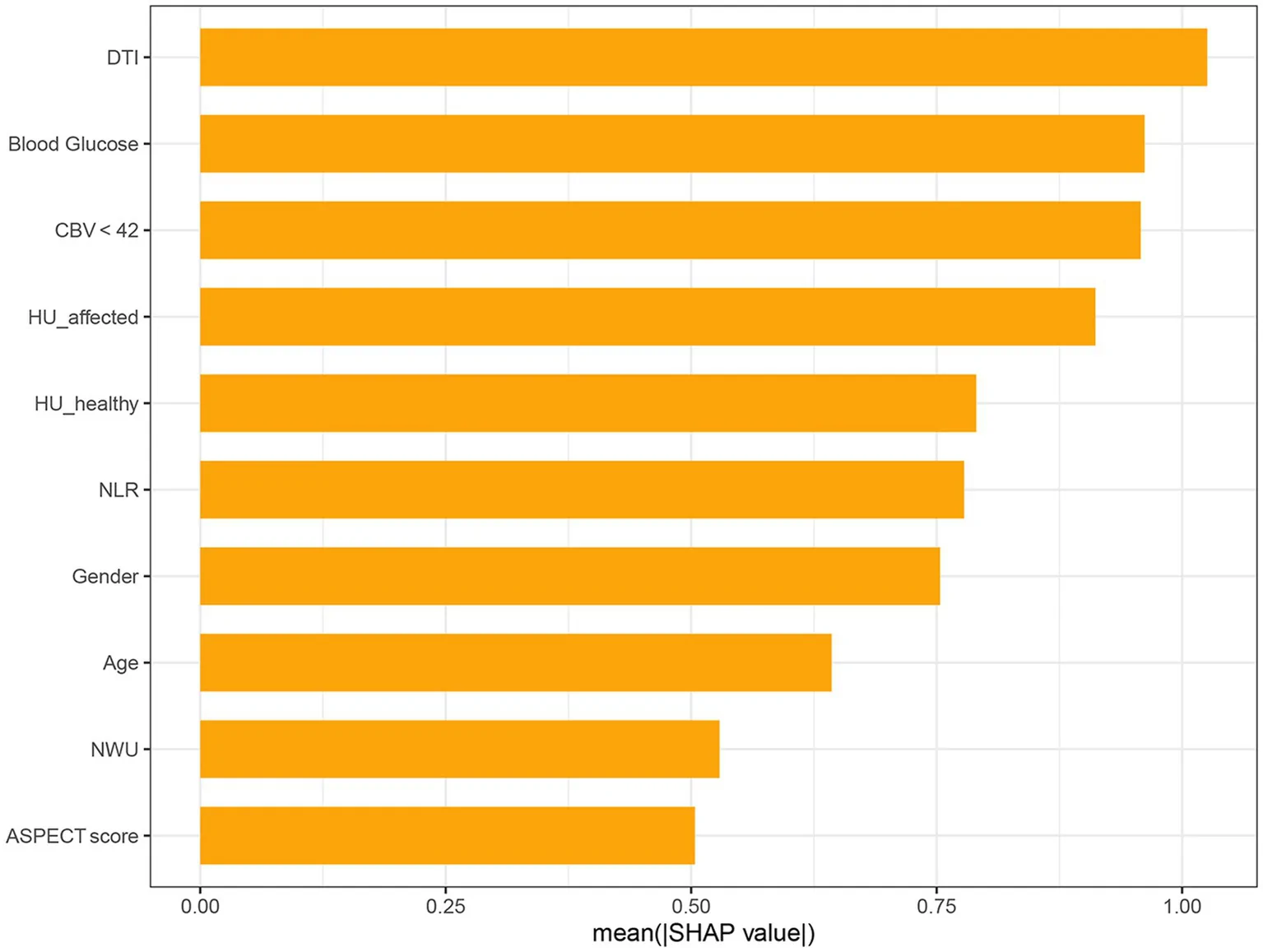
Mean absolute SHA*p* values of the ten predictors in the XGBoost model. DTI was the most influential predictor, followed by HU_affected, CBV < 42%, NLR, Blood Glucose, Age, Gender, ASPECT score, HU_healthy, and NWU.

To interpret the predictions of the XGBoost model, we calculated SHAP values for each feature in the validation set. The mean absolute SHAP values revealed that DTI was the most influential predictor, followed by HU_affected, CBV < 42%, NLR, Blood Glucose, Age, Gender, ASPECT score, HU_healthy, and NWU (Figure 4).

**Figure 5:**
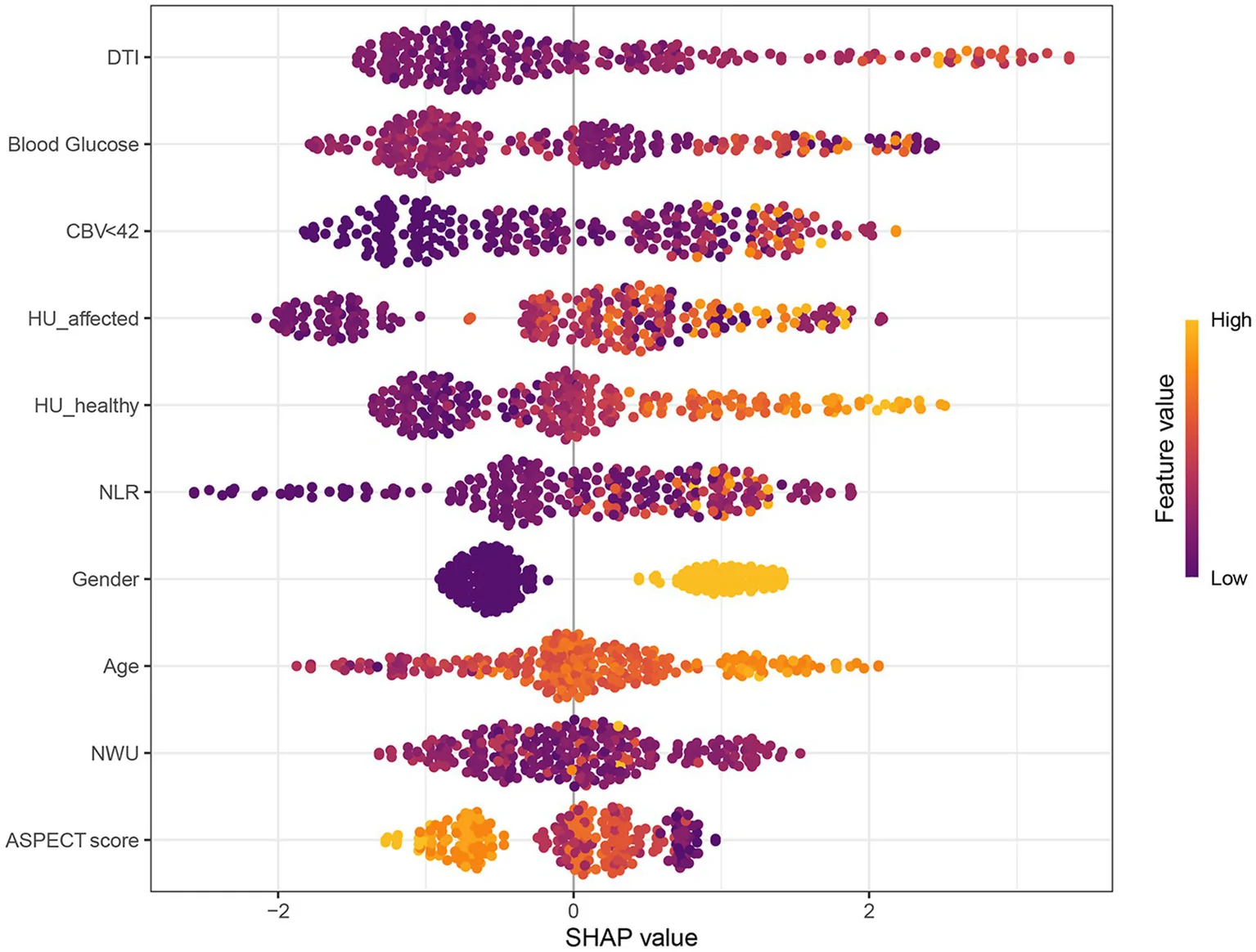
SHAP beeswarm summary plot of the nine predictors. Red indicates higher feature values; blue indicates lower values. Features are arranged in descending order of importance.

The beeswarm summary plot (Figure 5) demonstrated that higher values of DTI, HU_affected, CBV < 42%, NLR, Blood Glucose, Age, Gender (female, coded as 2), HU_healthy, NWU, and ASPECT score were generally associated with increased risk of poor prognosis (positive SHAP values), while lower values of these predictors were associated with decreased risk (negative SHAP values). Notably, for ASPECT score, higher values indicate less extensive early ischemic change; its association with positive SHAP values in this plot should be interpreted with caution, as this may reflect complex interactions with other imaging biomarkers in the model.

**Figure 6:**
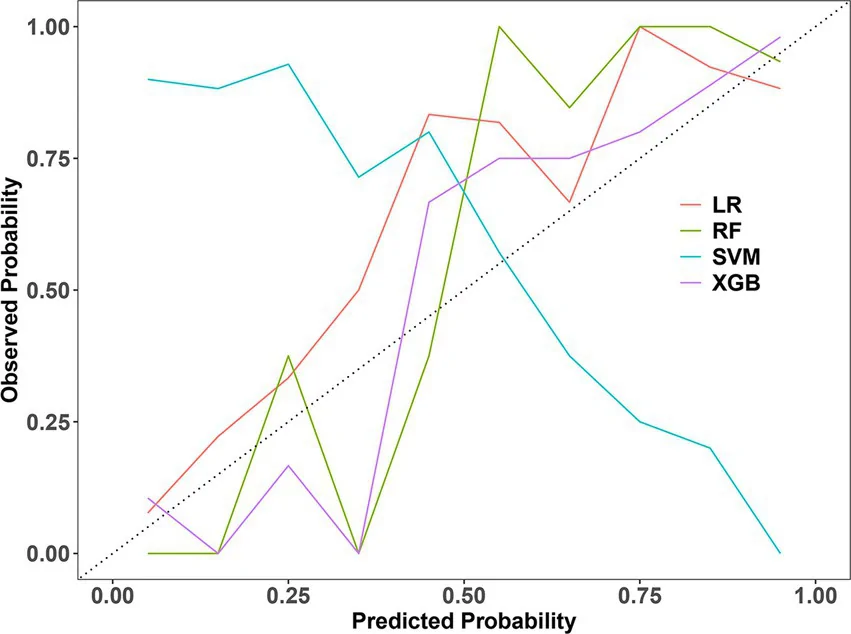
Calibration curves of the four prediction models in the validation set.

To visually assess the calibration of the four models, calibration curves were plotted in the validation set (Figure 6). The XGBoost model demonstrated the closest agreement with the 45° diagonal reference line, indicating excellent calibration. The Random Forest model also showed acceptable calibration, whereas Logistic Regression exhibited moderate overestimation at lower risk thresholds. Notably, the SVM curve deviated markedly from the reference line across most probability ranges, consistent with its poor Brier score (0.5339). These visual findings further support the clinical reliability of the XGBoost-based prediction model.

**Figure 7:**
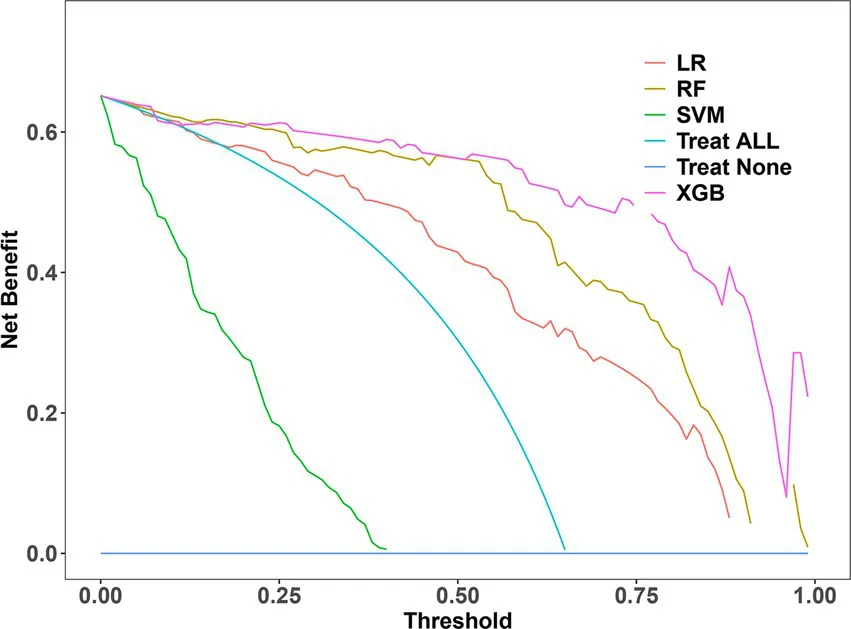
Decision curve analysis for the four prediction models in the validation set.

Decision curve analysis was performed to evaluate the clinical utility of the four models across threshold probabilities ranging from 0.01 to 0.60 (Figure 7). The XGBoost model consistently demonstrated the highest net benefit, followed by RF. Logistic regression showed moderate net benefit at lower threshold probabilities but declined as the threshold increased. Support vector machine exhibited poor clinical utility, with net benefit dropping below zero at threshold probabilities above approximately 0.40. Overall, XGBoost and RF outperformed the treat-all and treat-none strategies across most threshold probabilities, whereas SVM provided limited or no clinical value.

## 4 Discussion

In this retrospective cohort study, we developed and validated a machine learning-based model integrating multimodal data—clinical variables, laboratory markers, CT perfusion parameters, and quantitative CT density measurements—to predict 9-month poor functional outcome (mRS 3–6) in patients with acute ischemic stroke undergoing endovascular or surgical intervention. Among the four algorithms compared, the XGBoost model achieved the best overall performance in the independent validation set, with an AUC of 0.956 (95% CI 0.917–0.995), a sensitivity of 0.932, and excellent calibration (Brier score 0.0713, 95% CI 0.0438–0.1135). Through a two-step feature selection strategy combining LASSO regression with SHAP-based refinement, we identified a compact set of ten predictors, among which DTI (mean SHAP = 0.95), HU_affected (0.88), and CBV < 42% (0.82) emerged as the most influential. All predictors were derived from pretreatment admission data, and the model was therefore designed to provide baseline risk stratification prior to revascularization, rather than to evaluate the efficacy of the intervention itself.

Our results are consistent with and extend previous work on machine learning for stroke outcome prediction. A systematic review and meta-analysis reported that AI models achieve good predictive accuracy for ischemic stroke outcomes (9), and several recent studies have demonstrated the superiority of XGBoost over conventional logistic regression (10–12). Chung et al. developed an XGBoost model using only three clinical variables (age, fasting glucose, and NIHSS) to predict 3-month functional outcome, achieving an AUC of 0.8595 (10). Our model attained a higher AUC (0.956), which likely reflects the added value of integrating multimodal imaging and laboratory data beyond clinical variables alone. Similarly, Tong et al. reported a validation AUC of 0.93 for an interpretable XGBoost model in patients undergoing mechanical thrombectomy (12), a performance comparable to ours that supports the robustness of gradient-boosted trees in this setting. Unlike Ozkara et al. (13), who relied exclusively on imaging parameters, our framework incorporated clinical, laboratory, and imaging domains simultaneously, providing a more comprehensive prognostic profile. Importantly, whereas most prior studies focused on short-term (3-month) outcomes, our study addressed the less frequently examined 9-month functional outcome, thereby extending the temporal scope of machine learning-based stroke prognostication. This longer follow-up period may better capture the full trajectory of neurological recovery, as functional gains can continue beyond the first 3 months post-stroke. Moreover, 9-month outcome is increasingly recognized as a meaningful endpoint in stroke recovery research, reflecting more stable functional status than 3-month assessments.

A key finding of this study is that quantitative imaging parameters, along with the door-to-intervention interval, outweighed conventional clinical variables in prognostic importance. DTI emerged as the single most influential predictor in our SHAP analysis, underscoring the critical importance of timely reperfusion in determining long-term functional outcomes. HU_affected, which reflects early ischemic hypoattenuation and lesion water uptake—early imaging correlates of irreversible tissue injury and developing cerebral edema—ranked second in importance. Similarly, the volume of tissue with CBV < 42%, a marker of the irreversibly injured ischemic core, ranked third in importance. This finding aligns with Lakhani et al., who demonstrated that relative CBV < 42% was significantly associated with poor functional outcome at discharge in anterior circulation large vessel occlusion stroke (aOR = 0.97, *p* < 0.05) (14). Our results extend this observation by showing that the CBV-defined ischemic core retains its prognostic value for long-term (9-month) functional outcome. In addition, NWU—a quantitative estimate of early brain edema derived from non-contrast CT density—was retained as an independent predictor, in agreement with recent reports that NWU provides complementary prognostic information to conventional CT perfusion parameters (12, 15, 16). The concurrent inclusion of NWU and CBV < 42% in our model underscores that brain edema and infarct core represent two distinct but complementary pathological processes, both of which are independently captured by quantitative imaging.

Several non-imaging predictors also contributed meaningfully to the model. The door-to-intervention interval ranked first in importance, underscoring the well-established principle that “time is brain”: delays to reperfusion are associated with larger infarcts and worse functional outcomes (17–19). This finding carries direct clinical implications: efforts to streamline in-hospital stroke workflows and reduce door-to-intervention times may translate into measurable improvements in long-term prognosis. Admission blood glucose also emerged as an important predictor; stress hyperglycemia is known to aggravate ischemic injury and promote blood–brain barrier disruption, and has been associated with poor stroke outcome in both diabetic and non-diabetic patients (20). The neutrophil-to-lymphocyte ratio, a readily available marker of systemic inflammation, was likewise retained, consistent with prior evidence linking elevated NLR to stroke severity and unfavorable prognosis (9, 21). It is noteworthy, however, that NLR ranked relatively lower in feature importance once imaging parameters were included, suggesting that a substantial portion of the prognostic information carried by systemic inflammatory markers may be captured more directly by quantitative imaging of the ischemic lesion itself. Age, gender, and the ASPECT score—well-established prognostic factors—were also retained, further supporting the biological plausibility of the model.

The comparative analysis across algorithms offers additional insights. XGBoost outperformed logistic regression, reflecting its ability to model non-linear relationships and interactions among predictors without prespecified functional forms. In contrast, the SVM (RBF kernel) showed lower sensitivity (0.828) and poorer calibration (Brier score 0.5339) compared with tree-based models, indicating that the non-linear decision boundary of the RBF kernel was still insufficient to fully capture the complex structure of this dataset—further evidence of the inherently non-linear nature of stroke outcome prediction. SVM is also sensitive to feature scaling and may underperform in high-dimensional, sparse medical datasets, whereas tree-based models are scale-invariant and naturally handle non-linear partitions. Random forest achieved performance close to XGBoost, which is expected given that both are tree-based ensemble methods. Beyond discrimination, calibration is critical for clinical decision-making because predicted probabilities directly inform risk stratification. The XGBoost model achieved a Brier score of 0.0713—below the 0.10 threshold generally considered excellent—indicating that its predicted probabilities deviate from observed outcomes by an average of approximately 7 percentage points. It also followed the ideal diagonal most closely on the calibration plot. Decision curve analysis further demonstrated that XGBoost provided the highest net benefit across clinically relevant threshold probabilities (0.01–0.60), supporting its potential utility as a decision-support tool.

Our findings have several practical implications. First, all predictors are obtainable within 24 h of admission, allowing risk stratification early in the disease course—when decisions regarding early risk stratification and potential rehabilitation resource allocation are most consequential. Second, identifying patients at high risk of poor 9-month outcome could enable individualized rehabilitation strategies and more intensive surveillance for those who need it most. In particular, for patients identified by SHAP as high-risk despite being underestimated by conventional clinical judgment, the model may offer added value by reducing misclassification. Third, the use of SHAP explanations enhances model transparency: rather than acting as a “black box,” the model provides patient-specific, feature-level rationales for each prediction, which may foster clinician trust and facilitate clinical adoption. Finally, because the model relies on routinely acquired CT perfusion and laboratory data, it could be integrated into existing imaging post-processing workflows or electronic medical record systems with minimal additional burden.

The strengths of this study include the integration of multimodal data spanning clinical, laboratory, perfusion imaging, and density measurement domains. To our knowledge, this comprehensive approach remains relatively uncommon in the stroke outcome prediction literature. While many previous studies have relied on single-modality data (clinical variables or imaging parameters alone), our cross-modal integration framework demonstrates that combining these information sources provides incremental predictive value beyond any single modality. Additionally, we employed a rigorous two-step feature selection strategy that balances statistical parsimony with interpretability, yielding a clinically meaningful and computationally efficient model. We also established a comprehensive evaluation framework encompassing discrimination, calibration, and clinical utility (decision curve analysis), and focused on the relatively understudied 9-month functional outcome, which complements the predominantly 3-month focus of prior work.

Several limitations should be acknowledged. First, this was a retrospective, single-center study, which is subject to selection bias and limits generalizability. Second, the sample size was modest (*n* = 371), and no external validation cohort was available; although an independent internal validation set was used, external multicenter validation is essential before clinical deployment. Third, statistically significant differences in several baseline characteristics—including lymphocyte count, ASPECT score, NWU, Tmax > 10 s, and CBV < 42%—were observed between the training and validation sets. These differences were clinically modest and likely attributable to random sampling variation and multiple comparisons; nevertheless, such imbalances may have influenced performance estimates and warrant cautious interpretation (22). Fourth, our cohort comprised patients who underwent endovascular or surgical intervention, so the findings may not generalize to the broader AIS population managed conservatively. Fifth, NIHSS data were not available for analysis due to data quality concerns identified during retrospective preprocessing; this may limit the comparability of our findings with studies that include this established clinical scale. Sixth, several potentially important baseline variables—including stroke subtype (e.g., TOAST classification), prestroke medications, prior stroke history, and comorbidities—were not captured and may have confounded long-term outcomes. Treatment-related variables such as reperfusion success were also unavailable, as the model was designed for baseline risk stratification rather than post-treatment evaluation. Finally, HU measurements relied on manual ROI delineation, which is subject to inter-rater variability; automated segmentation approaches could mitigate this issue in future work. Despite these limitations, the variables included in our model already cover the range of multimodal data feasible in current clinical practice. The model’s strong performance in the independent validation set supports its potential clinical value, which awaits confirmation through further external validation.

Future studies should prioritize prospective, multicenter external validation; incorporate treatment-related variables such as reperfusion success; explore deep learning-based automated lesion segmentation to replace manual ROI delineation; and evaluate the clinical and health-economic impact of model-guided decision-making. As medical imaging analysis techniques continue to advance, such multimodal data-driven models are expected to achieve full automation and seamless integration into clinical workflows, offering precise and individualized prognostic assessments for stroke patients.

## 5 Conclusion

We developed an interpretable XGBoost-based model integrating multimodal clinical, laboratory, and imaging data that accurately predicted 9-month poor functional outcome in patients with acute ischemic stroke undergoing endovascular or surgical intervention. DTI, HU_affected, and CBV < 42% emerged as the dominant predictors, highlighting the prognostic importance of time-to-reperfusion and baseline tissue injury. Pending external validation, this model may serve as a practical decision-support tool for individualized risk stratification and rehabilitation planning.

## Data Availability

The original contributions presented in the study are included in the article/supplementary material, further inquiries can be directed to the corresponding author.
